# Bioremediation Monitoring

**DOI:** 10.1289/ehp.113-a444a

**Published:** 2005-07

**Authors:** Steven D. Aust

**Affiliations:** Chemistry and Biochemistry Department, Utah State University, Logan, UT, E-mail: sdaust@cc.usu.edu

In their article published in the February issue of *EHP*, Ganey and Boyd (2005) made some excellent points about the potential pitfalls of simply assaying for the disappearance of an environmental pollutant during or as a result of bioremediation. This is important because it would be wrong to leave a metabolite that might pose as much or even more risk then the original chemical of interest.

Ganey and Boyd (2005) used the bio-remediation of polychlorinated biphenyls (PCBs) as an example, which was an excellent choice. However, the subject of metabolism of the PCB bioremediation metabolites should also be considered. As chlorines are removed by bioremediation, the less-chlorinated products could be more readily metabolized by many species exposed to the bioremediated material. That is, less-heavily chlorinated products (or intermediates) of bioremediation may be less toxic because of shorter half-lives due to metabolism. This phenomenon can be exemplified by work we conducted years ago at Michigan State University. We showed that 3,4,3′,4′-tetrabromobiphenyl was less toxic than 3,4,5,3′,4′,5′-hexabromobiphenyl, even though it was bound at higher affinity by the dioxin receptors because it was more readily metabolized and eliminated (Millis et al. 1985).

Commercial preparations contain few or no strictly coplanar PCB or polybrominated biphenyl congeners. This fact does not seem to be appreciated, and the impression is sometimes given that those very toxic congeners are in the environment. In fact, the coplanar polyhalogenated biphenyls probably receive way too much attention, most likely because they were used rather extensively in research; however, they were used only as model toxic congeners. The synthesis of strictly coplanar halogenated biphenyls (i.e., 3,4,3′,4′-PCB) is much different from that of the commercial preparations (which was by simple halogenation of biphenyl). Phenyl is strongly *ortho*-*para* directing, leading to non-coplanar halogenated biphenyls. The initial *para* and/or *ortho* halogenation makes for an even stronger *ortho-para* directive. Thus, the major components will be non-coplanar halobiphenyls. Only very small amounts of single *ortho* halobiphenyls can be found in commercial mixtures, and these mixtures are quite ineffective in eliciting effects associated with binding by the dioxin receptor.
